# A PubMed-Wide Associational Study of Infectious Diseases

**DOI:** 10.1371/journal.pone.0009535

**Published:** 2010-03-10

**Authors:** Vitali Sintchenko, Stephen Anthony, Xuan-Hieu Phan, Frank Lin, Enrico W. Coiera

**Affiliations:** 1 Centre for Health Informatics, University of New South Wales, Sydney, New South Wales, Australia; 2 Centre for Infectious Diseases and Microbiology, Sydney Medical School, The University of Sydney, Sydney, New South Wales, Australia; 3 Institute of Clinical Pathology and Medical Research, Westmead Hospital, Sydney West Area Health Service, Sydney, New South Wales, Australia; St. Petersburg Pasteur Institute, Russian Federation

## Abstract

**Background:**

Computational discovery is playing an ever-greater role in supporting the processes of knowledge synthesis. A significant proportion of the more than 18 million manuscripts indexed in the PubMed database describe infectious disease syndromes and various infectious agents. This study is the first attempt to integrate online repositories of text-based publications and microbial genome databases in order to explore the dynamics of relationships between pathogens and infectious diseases.

**Methodology/Principal Findings:**

Herein we demonstrate how the knowledge space of infectious diseases can be computationally represented and quantified, and tracked over time. The knowledge space is explored by mapping of the infectious disease literature, looking at dynamics of literature deposition, zooming in from pathogen to genome level and searching for new associations. Syndromic signatures for different pathogens can be created to enable a new and clinically focussed reclassification of the microbial world. Examples of syndrome and pathogen networks illustrate how multilevel network representations of the relationships between infectious syndromes, pathogens and pathogen genomes can illuminate unexpected biological similarities in disease pathogenesis and epidemiology.

**Conclusions/Significance:**

This new approach based on text and data mining can support the discovery of previously hidden associations between diseases and microbial pathogens, clinically relevant reclassification of pathogenic microorganisms and accelerate the translational research enterprise.

## Introduction

The rapid accumulation of scientific information continues to challenge our capacity to synthesise a collective view of knowledge in different disciplines, and hinders discover of anomalies that are unexplained or not congruent with current theories [1], [2]. Uncovering such anomalies can be a potent catalyst to translational research and recently computational discovery has shown promise in supporting these processes of knowledge synthesis and anomaly detection [3]. Text mining the scientific literature offers an opportunity to discover new relationships currently hidden within individual publications, literally ‘joining the dots’ across interdisciplinary collections [4]. For example, our current understanding of disease phenomena is inherently multi-layered, including phenome, genome, proteome and biochemical pathways [5]. The systematic association of genes and phenotypes has been a fruitful approach in biomedical knowledge discovery [6], [7], [8] and several information retrieval systems have been proposed to assist researchers in the field of human genetics and basic microbiology [9]-[15]. Such systems rely on the mining of MEDLINE text [9], [10] or employ a similarity-based inference of disease genes [11], [12]. However, they focus primarily on either molecular level host-pathogen interactions [13]–[15] or pathway analysis [16] and do not link the characteristics of pathogens to clinical syndromes, which significantly limits their value for translational research. Others have undertaken unconstrained searches of this space, without reference to available biological knowledge that could direct the search for meaningful relationships [6].

In this study we develop approaches to find and quantify existing but hidden relationships between clinical syndromes and individual pathogens in order to detect meaningful associations across multiple scales. This knowledge involves different types of biomedical entities (e.g., genes, pathogens, drugs) and events (syndromes and diseases), which can be expressed using standard terms or as relationships among such objects [17]. We believe infectious diseases can act as an illustrative example of the broader potential for multi-scale text association studies in biology and translational research.

## Results

### Mapping the Infectious Disease Universe

**Figure 1:**
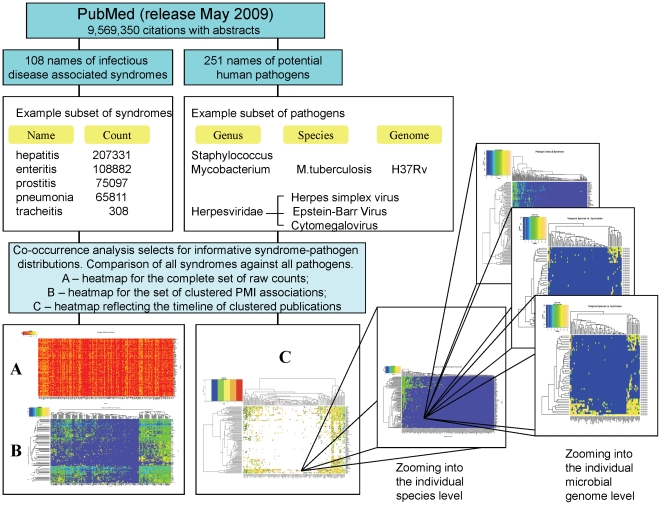
Systematic mining of publicly available biomedical text combines literature mining and the genome analysis of associate genes and phenotypes. Words describing infectious disease related syndromes, names of pathogens and names of individual genes obtained from [32], [33] and CMR database are retrieved from PubMed abstracts [31]. Syndrome-pathogen and microbial genus-syndrome word pairs with a point-wise mutual information (PMI) scores, respectively, greater than 20 and 5 suggestive of informative associations which are visualized as ‘heat maps’. (**A**) Initial representation of raw counts of PMI scores for all pairs of syndrome-genus name of a pathogen. (**B**) Preferential co-occurrence of specific syndromes and pathogen names is emphasized by hierarchical clustering. (**C**) ‘Doppler’ map of syndrome-pathogen associations revealing clusters of increased interest, which are judged by the rapid growth in the number of publications. An example of highly clustered pathogen Mycobacterium is then explored further by building the heat map of associations between syndromes and the list of individual *Mycobacterium* species, then by zooming into the heat map of associations between the list of syndromes and individual genes of *Mycobacterium tuberculosis* H37Rv. Individual genes not mentioned in the searched collection of abstracts were not included in the heatmap. Clusters of associated syndromes and pathogens include many previously known relationships.

**Figure 2:**
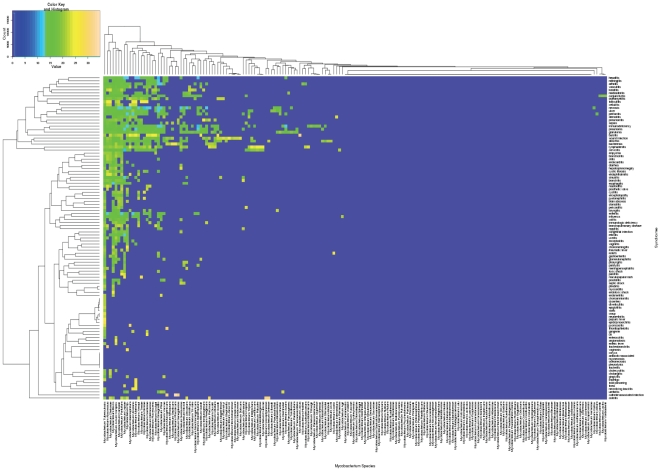
Associations between individual *Mycobacterium* species and infectious disease related syndromes.

A total of 12,631 pairs of infectious disease syndrome and pathogen names were identifiable in 589,694 individual articles (6.2% of the total pool of abstracts in NCBI PubMed [8]). The top twenty associations by frequency of co-occurrence and their strengths of association are reported in Table S1 (Supporting Online Material-SOM). Co-occurrence was measured by the frequency with which any two entities - pathogens, infectious disease syndromes, and so on - are found in the title or abstracts of individual published, peer-reviewed manuscripts stored in PubMed. Names were based upon a standardized hierarchy of pathogens and syndromes (see SOM for detail). The domain of infectious diseases was then represented as a 2x2 matrix of associations between pathogens and clinical syndromes (Fig. 1). Each line in this ‘heat map’ visualises the pattern of syndromes linked to a single microorganism, or the pattern of microorganisms linked to a syndrome of interest (Fig. 2 and Figures S1, S2). Evaluation of our text mining algorithms demonstrated high levels of retrieval accuracy (e.g., 100% recall, 89.3% precision and an F measure of 94.3 for syndrome-pathogen associations; Table S2).

### Dynamics of Literature Deposition

Temporal trends in the co-occurrence of syndromes and pathogens were explored (Fig. S3). Pathogen-syndrome associations were time-stamped by publication date to illuminate the rise and fall of topics in infectious disease research. While the landscape in the last decades of the 20^th^ century has been dominated by reports related to HIV and *Escherichia coli* (the “work horse of bacterial genomics”), new trends are emerging. Specifically, respiratory tract infections due to viruses and bacteria and sepsis, bacteremia and wound infections have been reported in PubMed with greater frequency in the last decade than any other topic apart from HIV-related research. Specific examples of ‘hot topics’ included clinical syndromes caused by coronaviruses and hepatitis associated with the Ureaplasma infection. Furthermore, the use of timelines facilitates the detection of emerging infectious disease syndromes from the PubMed corpus. For example, the co-occurrence frequency of West Nile virus and ‘encephalitis’ syndrome exceeded its expected historical levels in 2000 and peaked in 2006, and the pairing of terms “Chikungunya virus” and “arthritis” emerged in 2007 (Fig. S4).

### Zooming in from Pathogen to Genome Level

**Figure 3:**
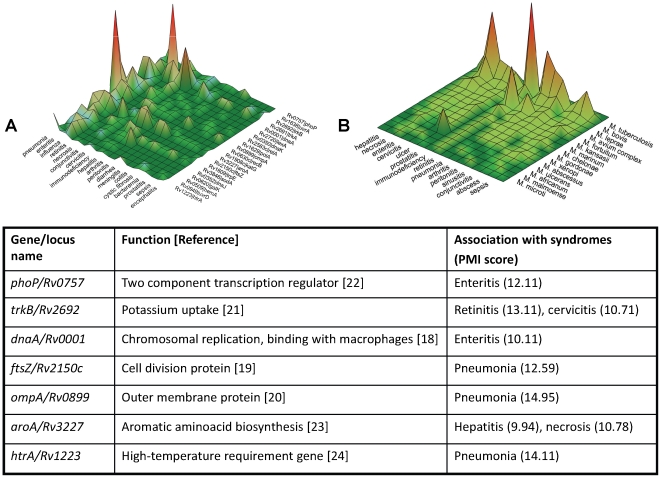
Landscapes of tuberculosis. Examples of species (**A**) and genome level (**B**) views of the tuberculosis landscapes with the selection of associations that suggest new hypotheses for testing. Peaks represent counts of co-occurrences of concepts. The table provides examples of associations between individual genes of *M. tuberculosis* H37Rv and syndromes (re)discovered in the experiment.

This associational space of clinical syndromes and pathogen genus names was next extended to visualise ‘syndrome-pathogen-species’ and ‘syndrome-pathogen-gene’ associations. 1119 and 757 gene names have been located in annotated genomes of *Mycobacterium tuberculosis* H37Rv and *Staphylococcus aureus* MRSA252, respectively (http://cmr.jcvi.org/tigr-scripts/CMR/CmrHomePage.cgi). Individual genes of *Staphylococcus aureus* were cited in 1890 abstracts or 10.8% of papers in which *S. aureus* was mentioned alongside infectious disease syndromes. Similarly, locus names and gene names of *Mycobacterium tuberculosis* were mentioned in abstracts of 340 articles or 7.3% of PubMed abstracts in which *M. tuberculosis* co-occurred with disease phenotypes. Figure 3 provides a snapshot of two levels of this associational landscape: the distribution of syndromes most frequently associated with different species of Mycobacteria and the selection of syndromes found to be linked to different genes in the *M.tuberculosis* H37Rv genome. These associations between individual genes and syndromes both recapitulate already known links but also generate new testable hypotheses that could reveal novel mechanisms of microbial virulence. For example, the role of molecular chaperones encoded by the *dnaA* gene in macrophage evasion has been reported after our experiment was completed [18]. Another gene identified in the study - the cell division protein gene (*fts*) - has been recently chosen as a new target for drug discovery [19]. These observations suggest further attention should be paid to the role of outer membrane protein A gene (*ompA*), - “a molecular Swiss army knife” [20], - in tuberculous pneumonia, as well as to the effects of the potassium uptake system, regulated by the *M.tuberculosis trkB* gene, in the pathogenesis of tuberculous retinitis (Fig. 3) [21]. Other examples of the knowledge rediscovery included associations between infectious disease syndromes and *phoP* [22], *aroA* [23] and *htrA* genes [24].

### Discovery of New Associations

**Figure 4:**
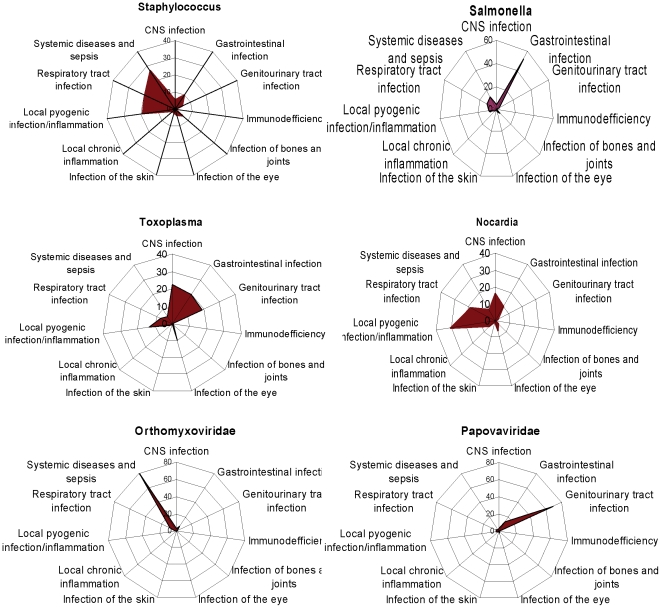
Syndromic signatures quantify differences between pathogens. Radar chart scale reflects the frequencies of co-occurrence of individual pathogen genus names and infectious disease syndromes.

**Figure 5:**
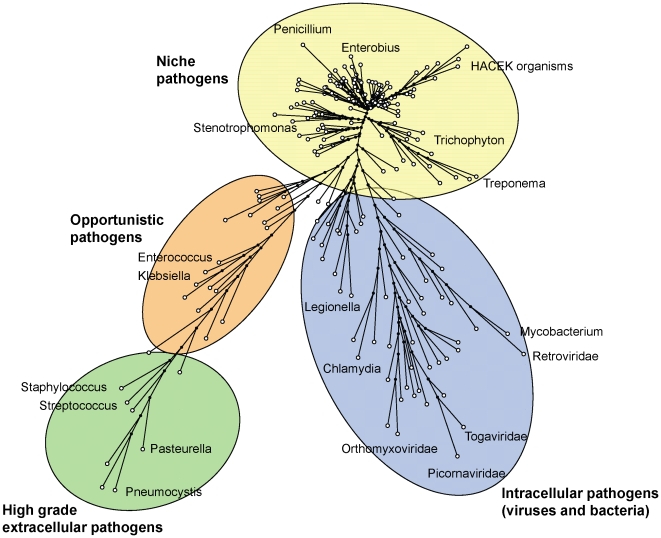
Clinically relevant reclassification of pathogens. Maximum parsimony tree represents the distance matrix of associated syndromic signatures.

The set of syndromes co-occurring with individual microorganisms can be thought of as a *disease signature* for each pathogen (Fig. 4). The diversity of such pathogen signatures may allow them to be used for syndromic surveillance of pathogens [17]. These relationships were identified using point-wise mutual information scores (PMI) and then used to construct a pathogen similarity tree, which has several interesting features (Fig. 5). A subset of bacteria including staphylococci, streptococci and Pasteurella together with Pneumocystis were clustered together reflecting their association with invasive disease. These high-grade extracellular pathogens appeared to neighbour opportunistic bacteria such as Klebsiella and Enterococci, which are characterised by a relatively low propensity to cause disease but a more aggressive behaviour in situations when they reach unprotected sites (blood, tissues) or when the host immune system is defective. In contrast, the majority of virulent viruses such as Retroviridae, Picornaviridae and Myxoviridae, along with Mycobacteria, Legionella and Chlamydia, were clustered with other pathogens characterised by intracellular mechanisms of microbial survival and attack. The virulence potential of individual pathogens appears proportional to the distance from the root of the tree (Fig. 5).

**Figure 6:**
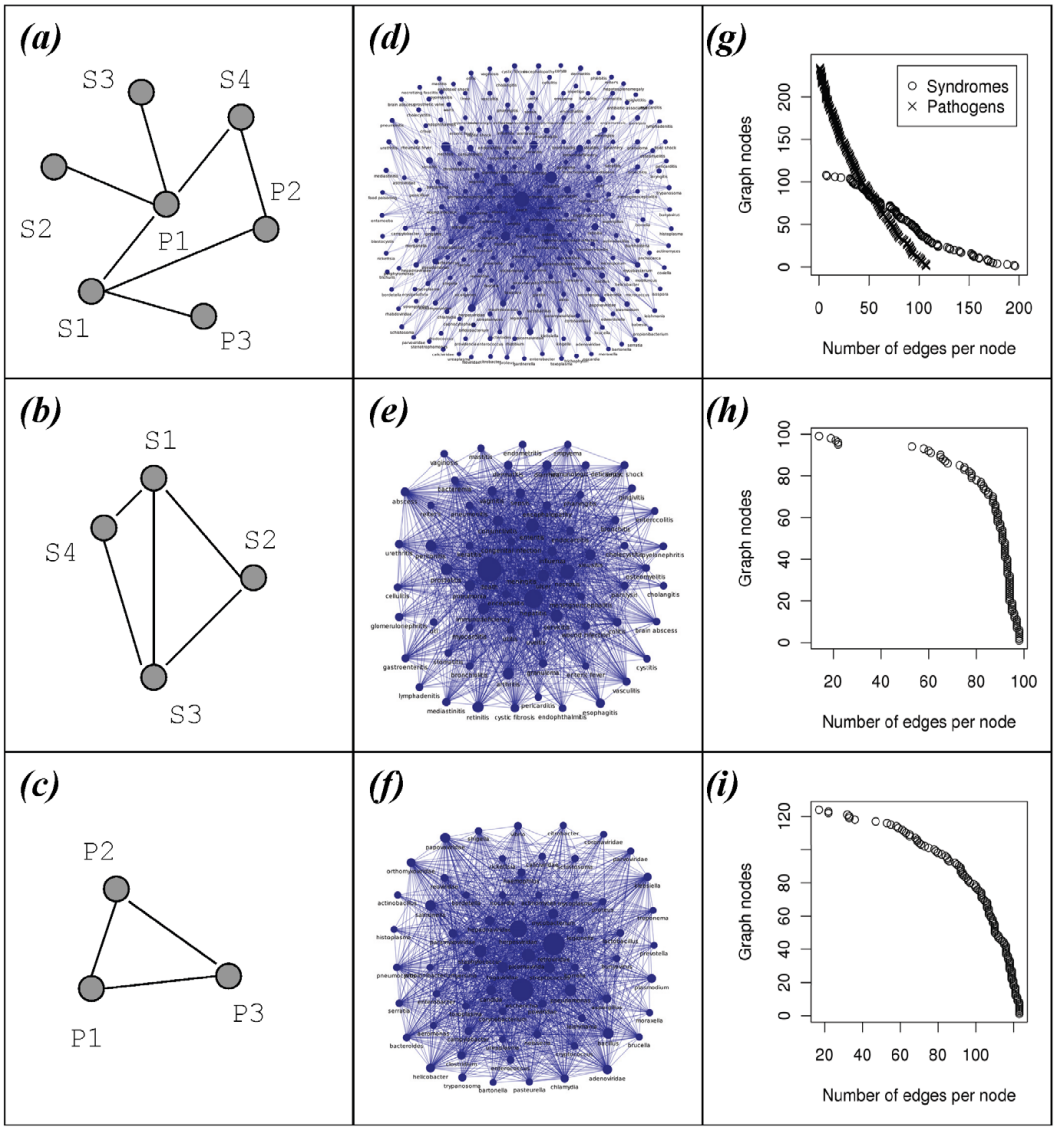
Associational network representation of syndrome-pathogen relationships. (*a*) Hypothetical network of syndromes and pathogens with edges representing the number of co-occurences in the text (mixed syndrome-pathogen network). (*b*) Hypothetical network of syndromes linking syndromes that co-occur with the same pathogens (syndrome only network); the length of the edge is inversely proportional to the number of shared pathogens. (*c*) Hypothetical network of pathogens linking pathogens that co-occur with the same syndromes (pathogen only network); the length of the edge is inversely proportional to the number of shared syndromes. Syndrome-pathogen (*d*), syndrome (*e*) and pathogen (*f*) networks built from text mining experiments; the size of a node represents the number of citations. The most highly connected nodes are in the middle. The edge per node distribution for each network is shown in respective graphs (*g, h, i*). Specific subsets of networks and lists of the most connected nodes can be found in SOM.

**Figure 7:**
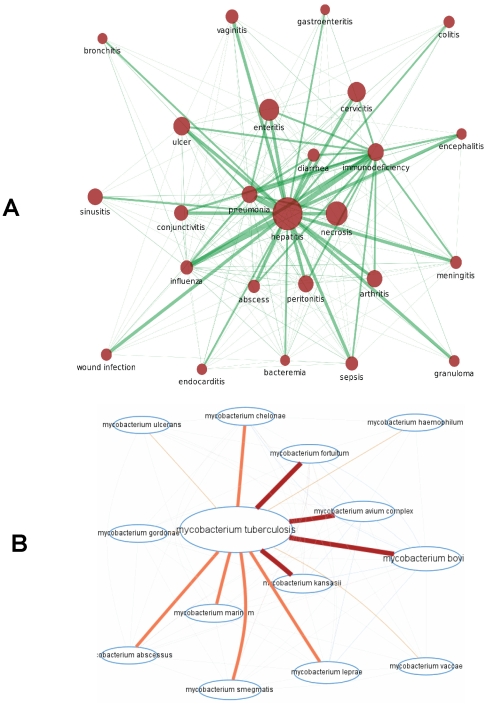
Associational networks of syndromes and pathogens. Examples of associational networks of syndromes (**A**), pathogens (**B**) and genes of an individual species (**C**). Node size depics the number of citations. Edge distance between two nodes is inversely proportional to the number of pathogens (A), syndromes (B and C) shared by concepts of nodes. The thickness of an edge is proportional to the normalised number of co-occurrences. Minimum number of co-citations with other pathogens for each entity in the network A to be included in this network was 15. The individual species gene associational networks (B) are presented using *M.tuberculosis* H37Rv genome as an example. Gene names and gene locus numbers are presented.

We also generated syndrome-pathogen, syndrome and pathogen networks to identify highly connected syndromes and pathogens (Fig. 6). The top 30 most connected nodes by syndrome and by pathogen are reported in Table S3. The four most connected syndromes and pathogens were, respectively, “pneumonia”, “enteritis”, and “peritonitis” and “abscess” as well as “Retroviridae”, “Staphylococcus”, “Herpesviridae” and “Streptococcus” (Table S3, and Fig. S5). Networks of pathogens at the species level, and networks of individual genes in microbial genomes linked to infectious disease syndromes, were also constructed (Fig. 7). For example, genes involved in DNA repair (e.g., *recA* (Rv2737)), synthesis of outer membrane proteins and drug resistance mechanisms (e.g., *gyrA* (Rv0006), *rpoB* (Rv0667)) were most frequently cited in relation to infectious disease syndromes.

## Discussion

We describe a new system that we expect to improve the discovery and assessment of infectious diseases, with broader potential for biomedicine. Rather than engaging in a ‘model-free’ approach to text mining adopted by others, where the frequencies of all words are analysed irrespective of their meaning, we have only mined those associations directly related to the discovery task at hand. The use of multilevel representations enables the construction of ‘zoomable’ associational maps with multiple views. Patterns for particular pathogens can be zeroed in on by moving from the initial high-level of microbial genus to the species taxonomy level, and subsequently onto the individual genome level (Fig. 1C).

To demonstrate the power of this approach we suggest a more clinically relevant alternative to the traditional evolutionary classification of pathogens, based upon their clinical syndrome associations. The initial metrics used to develop such a taxonomy may also be useful for building clinical risk assessment and decision support systems. Our approach readily separated pathogens capable of causing the broad spectrum of syndromes (e.g., *Staphylococcus spp*, *Streptococcus spp, Mycobacterium spp*) from microorganisms responsible for a limited range of conditions (*Arcanobacterium, Gemella, Wuchereria*). Interestingly, co-occurrence counts and PMI scores detected different phenomena with the latter identifying non-trivial associations. Examples of the most frequently detected associations include “hepatitis + Picornaviridae”, “ulcer + Helicobacter”, “neuroretinitis + Bartonella” (well known – a validation of the method), “epididymoorchitis + Brucella” (an unexpected finding confirmed by literature follow-up) (Table S1). Hierarchical clustering illuminates syndromes and specific pathogens that are more frequently associated with viral infections such as hepatitis, encephalopathy, myocarditis and uveitis.

This visualization approach thus permits the integration of different frames of reference to support hypothesis testing and generation. Such knowledge integration should help the search for strategic and tactical targets for scientific enquiry. For example, it can guide the prioritization of microbial sequencing. Currently, there are two schools of thought about the use of high-throughput gene sequencing in microbiology. One relies on the systematic sequencing of individual representatives for every phylogenetic node of the microbial Tree of Life [25]. The other argues that the focus should be switched from indiscriminate microbial analysis to the sequencing of classes of microbes of high relevance to human health, industry or the environment. However, no specific strategies for selecting such high-relevance species of microorganisms have been offered, despite the large publicly available datasets of microbial genomes. The accelerating pace of genomic sequencing and the increasing number of genes addressed in single studies and in high-throughput experimentation means that this corpus is growing. New approaches to extracting key findings and linking them to genes are therefore urgently needed.

A comparison of the distributions of highly cited pathogens in our study and fully sequenced microbial genomes reveals many gaps between the microbial genomes considered for full sequencing and those pathogens which are clinically relevant or cause high-burden diseases with epidemic potential (Fig. S6). It seems that geneticists focus either on pathogens with relatively short or well-characterized genomes such as viruses or *E. coli*. Viral genomes have attracted much sequencing effort with scores of different viruses representing Picornaviridae, Retroviridae and Caliciviridae already fully sequenced. The overrepresentation of viruses among the pathogens with completely sequenced genomes is likely to in part be a reflection of the relative ease of sequencing of them, given their genome size. Yet equally important pathogens representing *Neisseria, Staphylococci* and *Streptococci* and some epidemic parasites remain under-sequenced. The relative importance or degree of clinical and public health relevance of a pathogen can be expressed as a function of citation frequency (a proxy for importance) and of the relative frequency of syndromes co-occurring with this pathogen (Fig. S7). Such an information theoretic approach may have merit for prioritizing sequence analyses. However, confounding factors such technological advances that affect publication and molecular database volumes will add ‘noise’ to the analysis of temporal trends.

A key insight into computationally identifying nontrivial associations is that one has to consider one-to-many relationships when exploring biological entities. Such relationships can be captured and visualized by associational networks [26]. Network data structures are amenable to computational analyses which may help to uncover non-obvious properties of nodes and relationships between them. Networks of relationships suggest common mechanisms of disease between pathogens sharing common syndromes. Such analyses could provide new insights in the pathology of infectious diseases. Network topography can also imply biological similarity in disease epidemiology and is especially relevant for our understanding of polymicrobial infections. Therefore the indirect relationship and close proximity of Retroviridae with *Mycobacterium* nodes as well as Herpes simplex virus, *Staphylococcus* and Picornaviridae nodes in the network topology are of interest. It is likely that generating networks of microbial genes common to such clusters will offer specific insights about the rationale for higher-level syndrome-pathogen networks. An important capability of network representations is their capacity to relate entities of one dimension (e.g., syndromes) to entities from another dimension (pathogens) (see Fig. 6 and Fig. S4). Our approach explores the power of conducting large-scale association studies in text, and does not address finer detailed semantic issues such as negation, speculation, and context-dependent relations. Further work on the identification of semantic relationships should increase the effectiveness of text mining for hidden associations and potentially distinguish ‘negative’ associations. Natural language processing techniques such as semantic role labelling can identify predicates and their corresponding semantic arguments, leading to the uncovering of specific relations between entities, such as causation, expression, translation, and regulation [5], [8]. This level of semantic analysis should provide an opportunity to investigate more complex relations by employing inference techniques over the networked relationships that are discovered.

Two points are worthy of note. First, we have demonstrated the rediscovery of biomedical associations directly from publicly available repositories. Such explorations of “hidden public knowledge” have previously made predictions that were later confirmed by experimentation [27], [28]. Many applications exist for this approach, in fields ranging from biomedicine to systems biology, where significant gaps in knowledge still exist despite an abundance of data [13], [28], [29]. The future success of these applications depends heavily on the development of infectious disease ontologies that establish hierarchies for search entities and further improvements in the accuracy of text mining classifications. Second, the genomic layer provides a biological context for the visualisation and interpretation of data, and serves as a gateway to information stored in public databases (genome map viewers such as Gbrowse) [30], GMOD [31] and biochemical pathway databases such as KEGG [32], which can be visually explored from our maps. However, there are important differences between our approach and studies that have focused on finding pathways within metabolic networks using specific enzyme and substrate names. First, such studies often rely on mining full text articles and/or curated databases [6], [13], [33], whereas we only use article abstracts. Secondly, our approach couples associations between disease phenotypes and pathogens on three levels (disease-microbial genus, disease-microbial species and disease-microbial genome). Our system should encourage researchers to leave their ‘disease ghettoes’ and break interdisciplinary barriers by linking entities that have been historically studied by different sub-disciplines of virology, mycology, parasitology, mycobacteriology, immunology and dermatology, among others.

Several limitations and challenges to our global exploration of the collection of available papers in Medline should be acknowledged. First, literature-wide association studies are open to publication bias [8], [9], [28], [33] and our findings confirm the high proportion of papers addressing a limited number of high-visibility infectious diseases such as HIV and tuberculosis. Second, the importance of the quality of terminology lists cannot be overstated. In this study, we have deliberately narrowed our focus to common infectious disease syndromes avoiding entities describing the individual symptoms of diseases or pathological findings, such as ‘stridor’ or ‘epidermoplasia’, for example. Thus, we have potentially limited the quality of our search output. Increasing the complexity of terminology lists would further enhance the power of analyses. Third, the specificity of the names of syndromes may be lower than the specificity of names of genes or enzymes because of the nature of molecular and clinical data [28]. The relationship between a disease phenotype and a pathogen may sometimes be ambiguous as disease phenotypes often reflect universal pathobiology and the spectrum of clinical presentations at different time-points in the natural history of a disease. However, our findings support recent observations [6], [33] that such phenotypes can still be useful for text mining. It is likely that further classification of phenotypes enriched by new standard ontologies and disease outcome entities [34] may improve this perceived specificity of disease phenotypes. Equally, because related terms appear with such high frequencies, they should often be easily resolved because of their tendency to statistically cluster.

There is a growing demand for an integrated ‘helicopter view’ of research information to enable more targeted and balanced research priority assessment and monitoring. Techniques that capture diversity and illuminate commonalities across different domains are likely to enhance our capacity for hypothesis generation and integrative research. Might this process diminish the role of bench-top discoveries in the future? Quite the contrary: scientists may use processes such as these to help focus on interesting phenomena and explain their meaning more rapidly. Our findings should thus facilitate a system science approach to biomedicine.

## Materials and Methods

### Data Sources

The 2009 MEDLINE/PubMed Baseline Distribution [35] with 17,764,826 citations, which includes 9,569,350 citations with abstracts, was utilized as the data source. A list of syndromes associated with infectious diseases (108 items) was modified from the National Cancer Institute (NCI) Metathesaurus Taxonomy (http://nciterms.nci.nih.gov/NCIBrowser/Dictionary.do) and from the SNOMED-CT (see Table S4 for detail). Names of infectious diseases syndromes or diseases uniquely associated with specific pathogens such as malaria, tuberculosis, dengue, psittacosis etc were excluded from the list. A list of microbial pathogens was compiled from the NCBI GenBank taxonomy database [36] and indexes of two authoritative reference texts in clinical microbiology and infectious diseases [37], [38]. The pathogen list (Table S5) included facultative and opportunistic pathogens of humans representing viral, prokaryotic and eukaryotic facultative and opportunistic pathogens (241 items) [39]. Lists of gene names or genomic locus names were downloaded from files of fully sequenced genomes available from the Comprehensive Microbial Resource (CMR) (http://cmr.jcvi.org/tigr-scripts/CMR/CmrHomePage.cgi). We thus identified 1119 *Mycobacterium tuberculosis* genes that listed both a gene symbols and locus identifiers. For example, the *M. tuberculosis* H37Rv genome contained 1476 such gene symbols.

### Text Mining and Discovery of Associations

Each PubMed abstract was indexed for each mention of a syndrome, pathogen or gene name in our lists, using the full-text indexing capabilities of the Postgres Database Management system. The SPECIALIST lexicon [34] supported detection of variants in syndrome and pathogen nomenclature (e.g., M.tuberculosis; M. tuberculosis), spelling (e.g., hemolytic-uremic syndrome; haemolytic-uremic syndrome; diarrhoea; diarrhea etc) and clinical abbreviations (e.g., toxic shock syndrome toxin; tst; TSST; Methicillin resistant *Staphylococcus aureus*; (MRSA); Shiga-toxin Escherichia coli; STEC etc). Word stemming was employed to allow approximate matches and increase recall (e.g. “Black Creek Canal virus” matched variants such as “Black Creek Canal (BCC) virus” and “Black Creek Canal virus (BCCV)”).

Co-occurrence of biomedical terms was measured. The title and abstract recapitulate the content of articles and provide a robust proxy for the full text [40]. If *A* is the set of documents that refer to an entity *a*, and *B* is the set that include entity *b*, then *n(A∩B)* provides the frequency of co-occurrence [41]. The strength of association between any two concepts from the lists was measured by their point-wise mutual information (PMI), defined as the logarithm of the deviation between the observed frequency of the terms and of the expected frequency if they were independent [42], [43]:
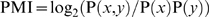



Relative frequency of citations for a pathogen reflects the weight of this pathogen in the corpus of knowledge and is calculated as the number of citations for this microorganism divided by the total number of citations for all pathogens. Relative frequency of co-occurrence with infectious disease related syndromes is calculated by dividing the number of individual pathogen co-occurrence with infectious disease related syndromes to the total number of citations for those syndromes.

### System Evaluation

Our concept indexing was evaluated by manual review of the labelling made by our system for 100 abstracts. This collection of abstracts contained 215 and 108 entities representing pathogens and syndromes, respectively. Standard information extraction metrics were then calculated including recall (R), precision (P), and F-measure [8], [43]. Recall is defined as the percentage of the system's correct hits or ‘true positives’ compared to all annotated items, including those that were missed or ‘false negatives’ (TP/(TP + FN)). Precision is defined as the percentage of true positives among all the extracted items, including spurious hits or ‘false positives’ (TP/(TP + FP)). The F-measure is a harmonic mean of R and P, defined as (2 x R x P/(R + P)). The closer the precision and recall are, the closer the F-measure score is to a standard average [44]. The recall, precision and F-measure scores are presented in Table S2.

### Knowledge Space Representation

The indexed abstracts were searched for each co-occurrence of a syndrome or pathogen, and each occurrence of each unique pairing counted [6], [8]. The search was repeated for Syndromes against entities representing different layers of knowledge: (1) Pathogen genus (e.g., Mycobacterium) (2) pathogen species names (e.g, *Mycobacterium tuberculosis*), (3) individual species genome (e.g., *Mycobacterium tuberculosis* H37Rv gene symbols), (4) viral pathogens (i.e. names of viral family and individual human viruses). The colour or “heat” of a particular pairing was based on the number of documents that contained the pair. Two versions of matrices were generated: one based on counts of co-occurrences and another one based on PMI scores. Either PMI or a logarithmic scale of counts was used. Hierarchical clustering was next employed using a standard Euclidean distance measure to calculate the dissimilarity between rows and columns [45]. Each row and column is initially assigned its own cluster. The algorithm successively merges the two most similar clusters based on the distance function until there is one single cluster.

Time course of publications was represented by Doppler graphs extracted from the PubMed collection which spanned the years 1913–2008 [33]. For each pair of syndrome and pathogen, we calculated the total number of abstracts that contained the pair for a given year, and its median year. We normalised the results with respect to the growth in annual publication count across PubMed by placing each pair's median year into one of five time periods, each containing one fifth of the cumulative infectious diseases corpus. The middle quintile corresponded to the median of all publications. The location of a median of a given pair was then assigned a hotter value if it was in the top 2 quintiles (postdating the median of all publications) or a cooler value if it was in the first two quintiles (predating the total median).

The patterns of syndromes and pathogens were aligned and maximum parsimony trees were built with BioNumerics 4.0 (Applied Maths, Sint-Martens-Latem, Belgium), which included the test for confidence intervals by bootstrapping (100 replicates).

### Associational Networks of Syndromes and Pathogens

The frequency counts of the co-occurrence of syndrome pathogen (or gene) pairs were used to generate different networks. Utilising an interactive tool [ http://purl.org/infectious/associations ] a number of associational networks were developed, emphasising the distance between syndromes as a function of their common pathogens, or the distance between pathogens, as a function of their common syndromes. In all views, node size represents the total frequency of the term appearing in a unique PubMed abstract.

In the syndrome network, the length of the edge connecting two syndromes is the inverse of the count of common pathogens. Edge thickness is the number of exclusive common documents. This allowed counting of abstracts that contained several different entities of syndromes and pathogens only once. Thickness was calculated as follows. Given two syndromes s1 and s2, and two viruses v1 and v2:
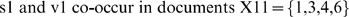


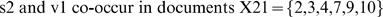


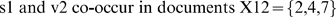


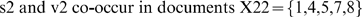



The following example illustrates how edge thickness (T) was calculated:



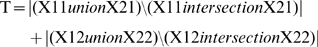


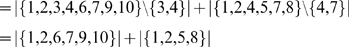


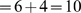



Heatmaps and plots utilised R [46], 2D networks were generated with Graphviz (graphviz.org) and graphs were produced using Cytoscape (version 2.6.1) [47].

## Supporting Information

Figure S1 The ‘heat map’ of raw frequency counts for all pathogens (X-axis) and syndromes (Y axis).
(0.10 MB TIF)

Figure S2 Associations between syndromes and pathogens. “Heat maps” display syndrome-pathogen association scores (scores greater than 0 are indicated; negative values are set to 0). All syndromes and all pathogens contributing to the respective cluster with at least one high-confidence association were considered.
(0.17 MB TIF)

Figure S3 Identification of changes in the publication rates by time stamping of co-occurrences (‘Doppler effects’).
(0.30 MB TIF)

Figure S4 Detection of the emerging “pathogen + syndrome” associations. This graph illustrates the increase of “West Nile virus” + “encephalitis” co-occurrence in 2000 which peaked in 2004–2007.
(0.07 MB DOC)

Figure S5 Associational networks of pathogens co-occurring with infectious diseases syndromes (A - sepsis; B - encephalitis). The size of each node is proportional to a number of citations. Minimum number of co-citations with other pathogens for each entity to be included in the network is 15.
(0.46 MB TIF)

Figure S6 Relative relevance of pathogens estimated by the frequency of citations. Relative frequency of citations for a pathogen reflects the weight of this pathogen in the corpus of knowledge and is calculated as the number of citations for this microorganism divided by the total number of citations for all pathogens. Relative frequency of co-occurrence with infectious disease related syndromes is calculated by dividing the number of individual pathogen co-occurrence with infectious disease related syndromes to the total number of citations for those syndromes.
(0.07 MB DOC)

Figure S7 Clinical and public health relevance of the subset of frequently cited pathogens. The size of each bubble is proportional to the number of fully sequenced genomes in the respective microbial genus or viral family (24). Relative frequency of citations for a pathogen reflects the weight of this pathogen in the corpus of knowledge and is calculated as the number of citations for this microorganism divided by the total number of citations for all pathogens. Relative frequency of co-occurrence with infectious disease related syndromes is calculated by dividing the number of individual pathogen co-occurrence with infectious disease related syndromes to the total number of citations for those syndromes.
(0.17 MB TIF)

Table S1 Top 20 “pathogen-syndrome” associations by the strength of association.
(0.05 MB DOC)

Table S2 Performance scores on entities and relations in blind set (100 abstracts).
(0.03 MB DOC)

Table S3 Top 30 most connected nodes by syndrome and by pathogen.
(0.08 MB DOC)

Table S4 List of syndromes used in the study.
(0.03 MB DOC)

Table S5 List of pathogen names used in the study.
(0.05 MB DOC)
