# Supplementary figures and images for: A PubMed-Wide Associational Study of Infectious Diseases
Source: PLoS One. 2010 Mar 10;5(3):e9535. doi: 10.1371/journal.pone.0009535 (PMC2835740; doi:10.1371/journal.pone.0009535)

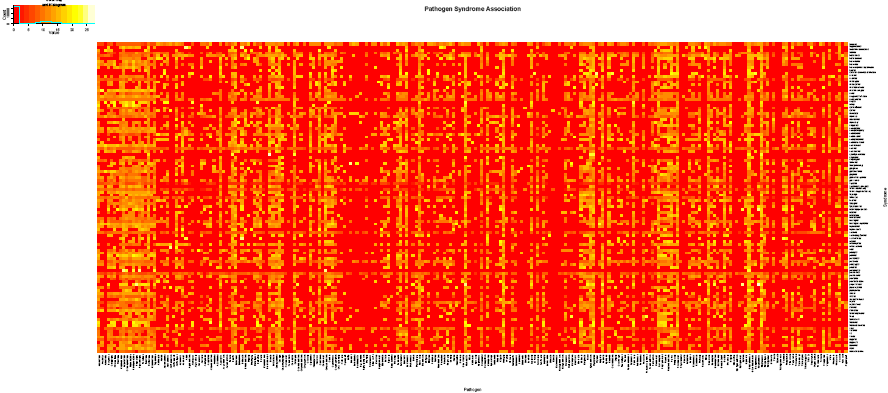

Supplement: Figure S1 — The ‘heat map’ of raw frequency counts for all pathogens (X-axis) and syndromes (Y axis). (0.10 MB TIF) [file pone.0009535.s001.tif]

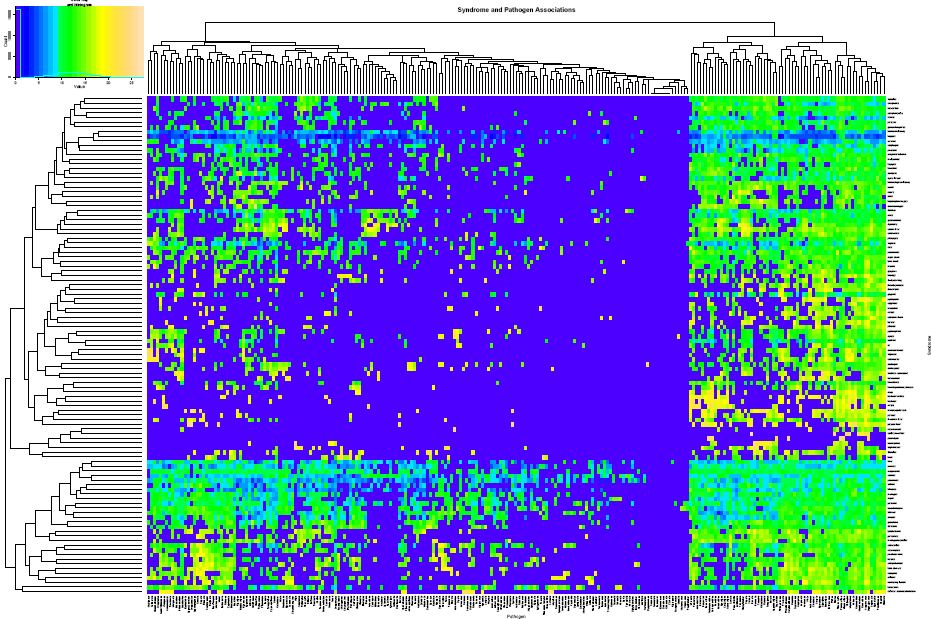

Supplement: Figure S2 — Associations between syndromes and pathogens. “Heat maps” display syndrome-pathogen association scores (scores greater than 0 are indicated; negative values are set to 0). All syndromes and all pathogens contributing to the respective cluster with at least one high-confidence association were considered. (0.17 MB TIF) [file pone.0009535.s002.tif]

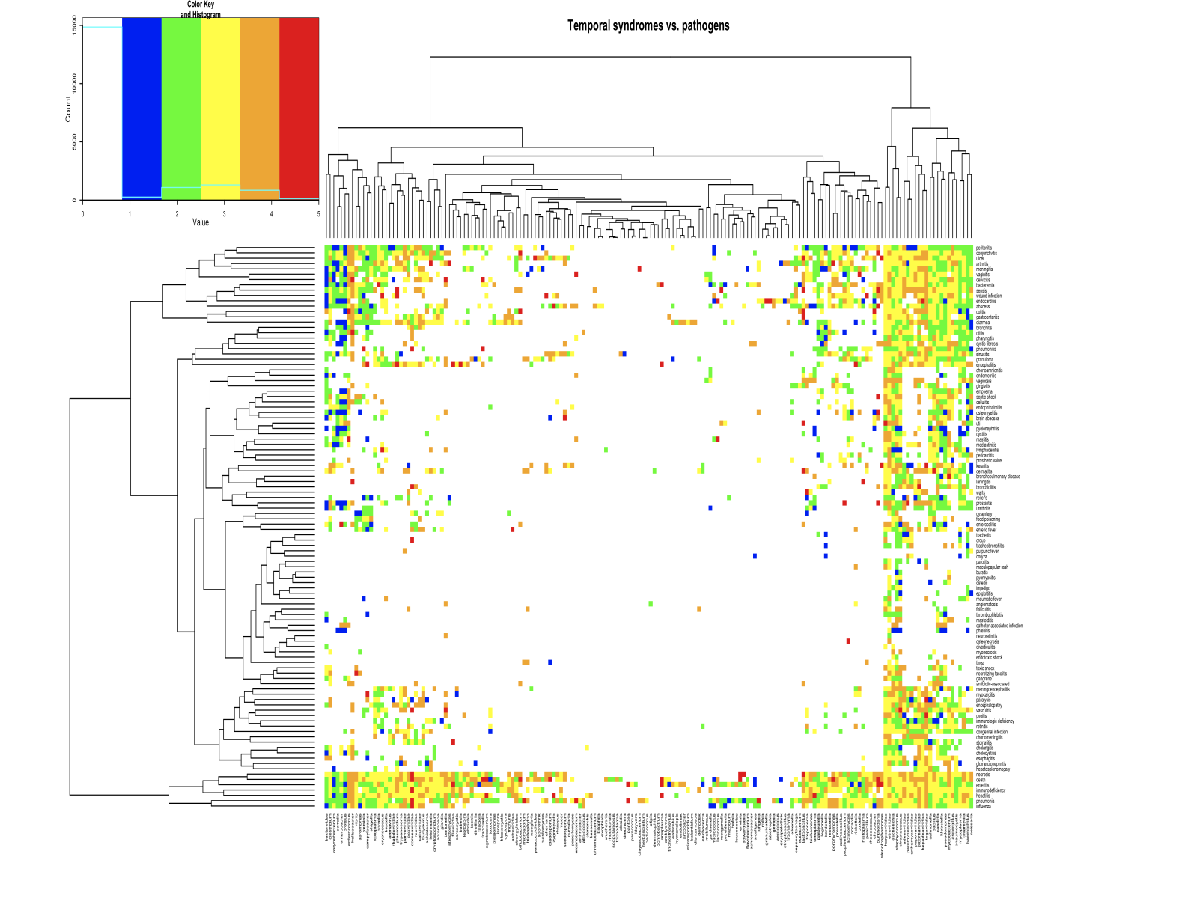

Supplement: Figure S3 — Identification of changes in the publication rates by time stamping of co-occurrences (‘Doppler effects’). (0.30 MB TIF) [file pone.0009535.s003.tif]

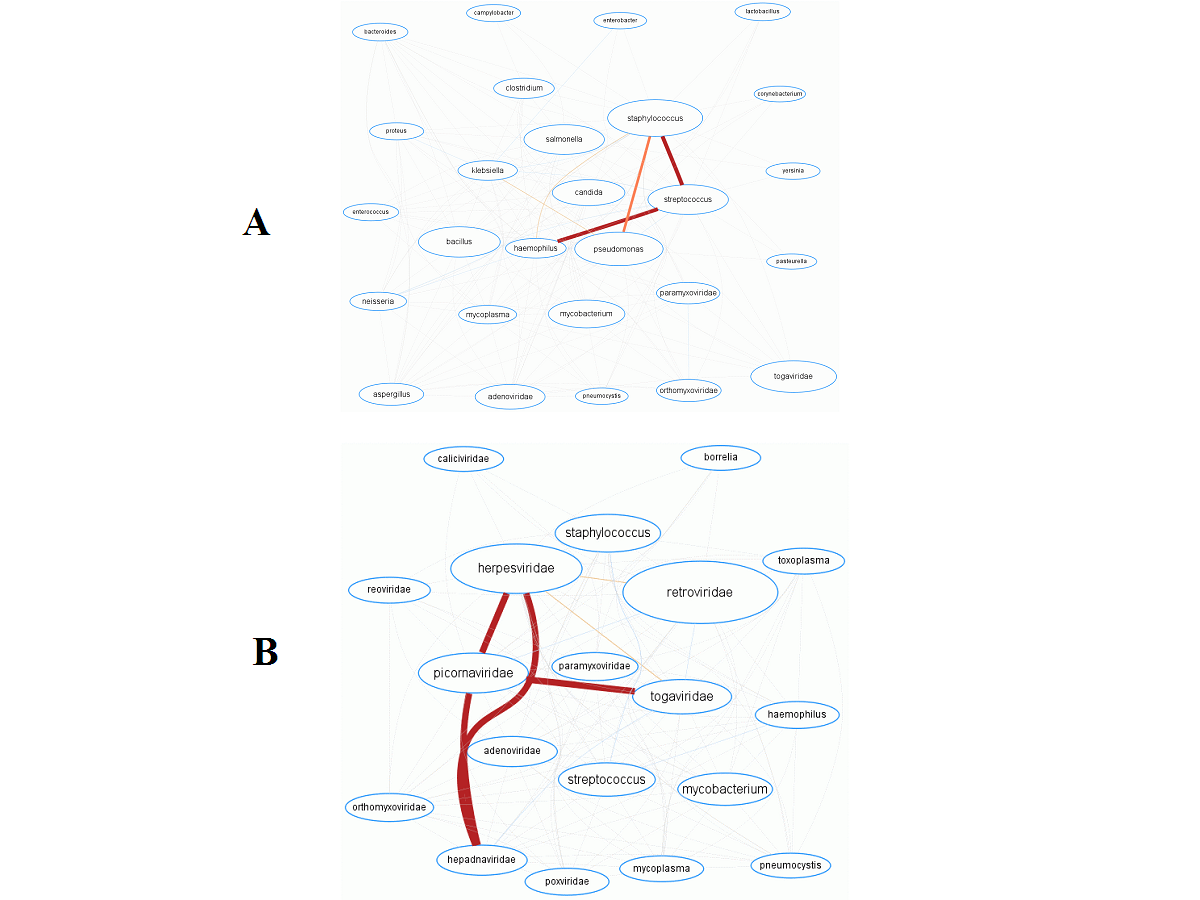

Supplement: Figure S5 — Associational networks of pathogens co-occurring with infectious diseases syndromes (A - sepsis; B - encephalitis). The size of each node is proportional to a number of citations. Minimum number of co-citations with other pathogens for each entity to be included in the network is 15. (0.46 MB TIF) [file pone.0009535.s005.tif]

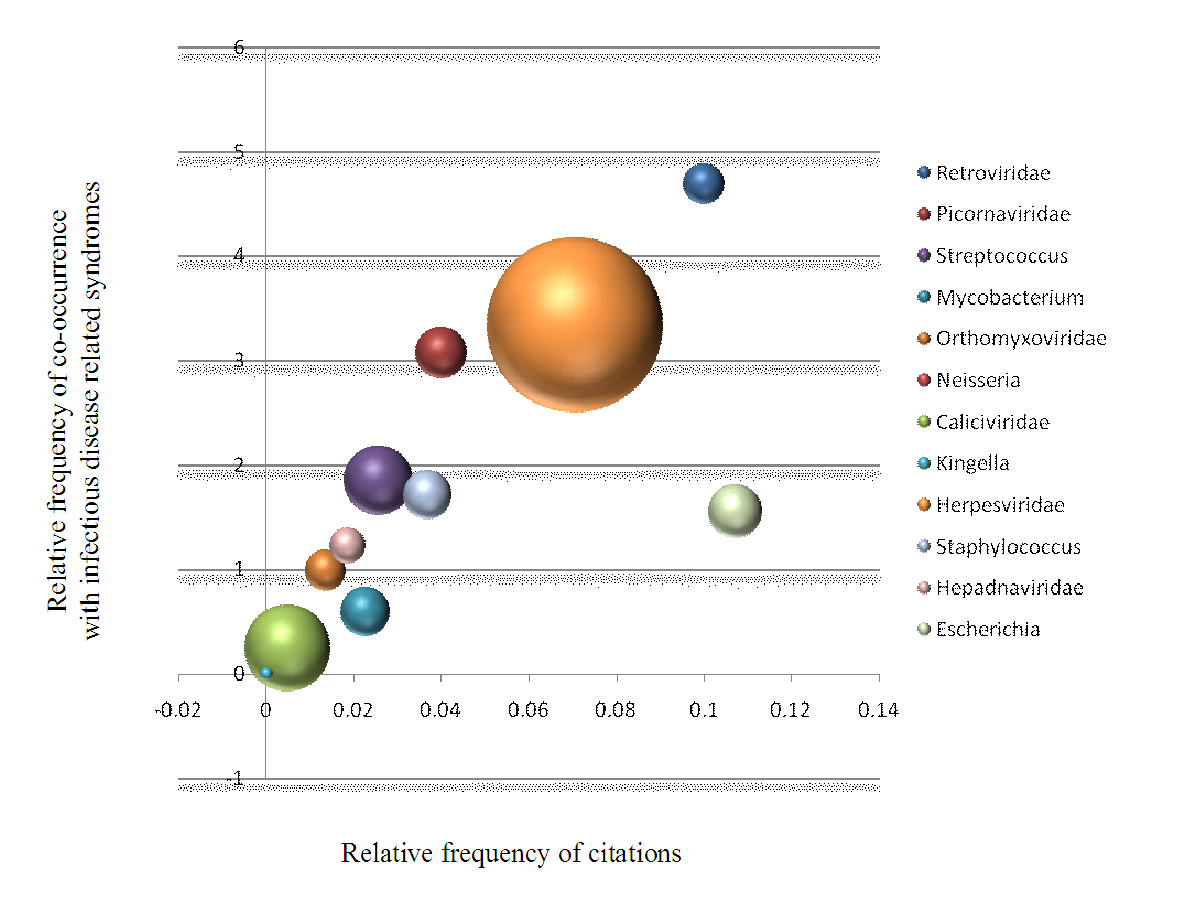

Supplement: Figure S7 — Clinical and public health relevance of the subset of frequently cited pathogens. The size of each bubble is proportional to the number of fully sequenced genomes in the respective microbial genus or viral family (24). Relative frequency of citations for a pathogen reflects the weight of this pathogen in the corpus of knowledge and is calculated as the number of citations for this microorganism divided by the total number of citations for all pathogens. Relative frequency of co-occurrence with infectious disease related syndromes is calculated by dividing the number of individual pathogen co-occurrence with infectious disease related syndromes to the total number of citations for those syndromes. (0.17 MB TIF) [file pone.0009535.s007.tif]
