# Supplementary material for: A PubMed-Wide Associational Study of Infectious Diseases
Source: PLoS One. 2010 Mar 10;5(3):e9535. doi: 10.1371/journal.pone.0009535 (PMC2835740; doi:10.1371/journal.pone.0009535)
Supplement: Table S1 — Top 20 “pathogen-syndrome” associations by the strength of association. (0.05 MB DOC) [file pone.0009535.s008.doc]

**Table S1.** Top 20 “pathogen-syndrome” associations by the strength of association

| **Rank** | **Pathogen-syndrome association** | **PMI score** |
| --- | --- | --- |
| 1 | "Brucella" + "epididymoorchitis" | 27.56 |
| 2 | "Streptobacillus" + "purpuric fever" | 26.52 |
| 3 | "Afipia" + "angiomatosis" | 25.45 |
| 4 | "Corynebacterium" + "pleurodynia" | 25.41 |
| 5 | "Bartonella" + "neuroretinitis" | 25.20 |
| 6 | "Spirillum" + "purpuric fever" | 25.15 |
| 7 | "Bartonella" + "angiomatosis" | 25.12 |
| 8 | "Streptobacillus" + "maculopapular rash" | 25.12 |
| 9 | "Picornaviridae" + "pleurodynia" | 24.86 |
| 10 | "Myroides" + "catheter-associated infection" | 24.74 |
| 11 | "Herpesviridae" + "pleurodynia" | 24.34 |
| 12 | "Demodex" + "folliculitis" | 24.33 |
| 13 | "Actinobaculum" + "osteonecrosis" | 24.21 |
| 14 | "Parvoviridae" + "purpuric fever" | 23.97 |
| 15 | "Haemophilus" + "purpuric fever" | 23.91 |
| 16 | "Afipia" + "neuroretinitis" | 23.85 |
| 17 | "Stomatococcus" + "catheter-associated infection" | 23.83 |
| 18 | "Mobiluncus" + "vaginosis" | 23.76 |
| 19 | "Leifsonia" + "catheter-associated infection" | 23.74 |
| 20 | "Mycoplasma" + "epididymoorchitis" | 23.74 |
