# Supplementary material for: A PubMed-Wide Associational Study of Infectious Diseases
Source: PLoS One. 2010 Mar 10;5(3):e9535. doi: 10.1371/journal.pone.0009535 (PMC2835740; doi:10.1371/journal.pone.0009535)
Supplement: Table S2 — Performance scores on entities and relations in blind set (100 abstracts). (0.03 MB DOC) [file pone.0009535.s009.doc]

**Table S2.** Performance scores on entities and relations in blind set (100 abstracts)

| **Entity** | **Recall** | **Precision** | **F measure** |
| --- | --- | --- | --- |
| Pathogen (215 entities) | 100 | 100 | 100.0 |
| Syndrome (108 entities) | 100 | 97.4 | 98.7 |
| Pathogen-syndrome  co-occurrence | 100 | 89.3 | 94.3 |
| Gene | 100 | 73.1 | 84.5 |
