# Supplementary material for: A PubMed-Wide Associational Study of Infectious Diseases
Source: PLoS One. 2010 Mar 10;5(3):e9535. doi: 10.1371/journal.pone.0009535 (PMC2835740; doi:10.1371/journal.pone.0009535)
Supplement: Table S3 — Top 30 most connected nodes by syndrome and by pathogen. (0.08 MB DOC) [file pone.0009535.s010.doc]

**Table S3.** Top 30 most connected nodes by syndrome and by pathogen

| **Rank** | **Syndromes** | | **Pathogens** | |
| --- | --- | --- | --- | --- |
|  | **Syndrome name** | **Total number of edges** | **Pathogen name*** | **Total number of edges** |
| 1 | pneumonia | 196 | Retroviridae | 107 |
| 2 | enteritis | 195 | Staphylococcus | 107 |
| 3 | peritonitis | 178 | Herpesviridae | 107 |
| 4 | abscess | 174 | Streptococcus | 106 |
| 5 | ulcer | 172 | Escherichia | 103 |
| 6 | immunodeficiency | 169 | Pseudomonas | 103 |
| 7 | conjuctivitis | 169 | Picornaviridae | 102 |
| 8 | necrosis | 168 | Mycobacterium | 102 |
| 9 | bacteremia | 160 | Candida | 102 |
| 10 | hepatitis | 160 | Salmonella | 101 |
| 11 | influenza | 155 | Klebsiella | 100 |
| 12 | meningitis | 154 | Bacillus | 98 |
| 13 | sepsis | 154 | Haemophilus | 98 |
| 14 | wound infection | 153 | Mycoplasma | 95 |
| 15 | diarrhea | 152 | Clostridium | 93 |
| 16 | sinusitis | 142 | Chlamydia | 93 |
| 17 | arthritis | 141 | Pneumocystis | 93 |
| 18 | endocarditis | 140 | Neisseria | 93 |
| 19 | granuloma | 135 | Togaviridae | 92 |
| 20 | cervicitis | 131 | Aspergillus | 92 |
| 21 | dermatitis | 130 | Paramyxoviridae | 91 |
| 22 | vaginitis | 128 | Proteus | 91 |
| 23 | osteomyelitis | 119 | Corynebacterium | 91 |
| 24 | congenital infection | 119 | Enterococcus | 90 |
| 25 | pneumonitis | 118 | Enterobacter | 90 |
| 26 | otitis | 118 | Orthomyxoviridae | 88 |
| 27 | keratitis | 113 | Bacteroides | 88 |
| 28 | gastroenteritis | 113 | Serratia | 88 |
| 29 | colitis | 111 | Adenoviridae | 88 |
| 30 | cellulitis | 109 | Hepadnaviridae | 85 |

Note: * Genus for prokaryotic and eukaryotic microorganisms and viral family’s name for pathogenic viruses
