# Supplementary material for: A PubMed-Wide Associational Study of Infectious Diseases
Source: PLoS One. 2010 Mar 10;5(3):e9535. doi: 10.1371/journal.pone.0009535 (PMC2835740; doi:10.1371/journal.pone.0009535)
Supplement: Table S4 — List of syndromes used in the study. (0.03 MB DOC) [file pone.0009535.s011.doc]

**Table S4.** List of syndromes used in the study

abscess

angiomatosis

antibiotic-associated

arthritis

bacteremia

brain abscess

bronchiolitis

bronchitis

bronchopulmonary disease

bursitis

catheter-associated infection

cellulitis

cervicitis

cholangitis

cholecystitis

chorioamnionitis

choriomeningitis

colitis

congenital infection

conjunctivitis

coryza

croup

cystic fibrosis

cystitis

death

dermatitis

diarrhea

diverticulitis

dysentery

empyema

encephalitis

encephalopathy

endocarditis

endometritis

endophthalmitis

endotoxic shock

enteric fever

enteritis

enterocolitis

epididymoorchitis

epiglottitis

esophagitis

folliculitis

food poisoning

gangrene

gastroenteritis

gingivitis

glomerulonephritis

granuloma

hepatitis

hepatosplenomegaly

immunodeficiency

Immunologic deficiency

impetigo

influenza

keratitis

laryngitis

lymphadenitis

maculopapular rash

mastitis

mastoiditis

mediastinitis

meningitis

meningoencephalitis

myocarditis

myonecrosis

necrosis

necrotizing fasciitis

neuroretinitis

osteitis

osteomyelitis

osteonecrosis

otitis

paralysis

parotitis

pericarditis

peritonitis

pharyngitis

phlebitis

pleurodynia

prostatitis

pneumonia

pneumonitis

prosthetic valve

purpuric fever

pyelonephritis

pyomyositis

reiter's

retinitis

rheumatic fever

sepsis

septic shock

sinusitis

stomatitis

tinea

toxic shock

tracheobronchitis

tracheitis

thrombophlebitis

ulcer

urethritis

uti

uveitis

vaginitis

vaginosis

vasculitis

warts

wound infection
