# Supplementary material for: A PubMed-Wide Associational Study of Infectious Diseases
Source: PLoS One. 2010 Mar 10;5(3):e9535. doi: 10.1371/journal.pone.0009535 (PMC2835740; doi:10.1371/journal.pone.0009535)
Supplement: Table S5 — List of pathogen names used in the study. (0.05 MB DOC) [file pone.0009535.s012.doc]

**Table S5.** List of pathogen names used in the study

Abiotrophia

Absidia

Achromobacter

Acinetobacter

Actinobacillus

Actinobaculum

Actinomadura

Actinomyces

Aerococcus

Aeromonas

Afipia

Agrobacterium

Alcaligenes

Alishewanella

Alloiococcus

Alternaria

Amblyomma

Anaplasma

Arcanobacterium

Arcobacter

Arthrobacter

Aspergillus

Aureobacterium

Aureobasidium

Bordetella

Babesia

Bacillus

Bartonella

Bacteroides

Balantidium

Bifidobacterium

Bilophila

Blastocystis

Blastomyces

Borrelia

Brevibacterium

Brevundimonas

Brucella

Burkholderia

Campylobacter

Candida

Capnocytophaga

Cellulomonas

Chaetomium

Chilomastix

Chlamydia

Chromobacterium

Chryseobacterium

Chrysosporium

Circinella

Citrobacter

Cladosporium

Clonorchis

Clostridium

Comamonas

Corynebacterium

Coxiella

Cryptococcus

Cryptosporidium

Curvularia

Cyclospora

Cunninghamella

Delftia

Demodex

Dermabacter

Dermatophilus

Dientamoeba

Diphyllobothrium

Dirofilaria

Dolosicoccus

Dracunculus

Drechslera

Dysgonomonas

Edwardsiella

Ehrlichia

Eikenella

Empedobacter

Endolimax

Entamoeba

Enterobacter

Enterobius

Enterococcus

Eremococcus

Erwinia

Erysipelothrix

Escherichia

Eubacterium

Ewingella

Exophiala

Facklamia

Fasciola

Flavobacterium

Fonsecaea

Francisella

Gardnerella

Gemella

Giardia

Globicatella

Gordonia

Granulicatella

Haemophilus

Hafnia

Halomonas

Hansenula

Helcococcus

Helicobacter

Herbaspirillum

Histoplasma

Hymenolepis

Iodamoeba

Isospora

Kingella

Klebsiella

Kluyvera

Koserella

Lactobacillus

Lactococcus

Laribacter

Latrodectus

Lautropia

Leclercia

Legionella

Leifsonia

Leishmania

Leminorella

Leptospira

Leuconostoc

Listonella

Massilia

Melissococcus

Methylobacterium

Microbacterium

Micrococcus

Microsporidium

Microsporum

Mobiluncus

Moellerella

Moraxella

Morganella

Mucor

Mycobacterium

Mycoplasma

Myroides

Naegleria

Neisseria

Nocardia

Nigrospora

Ochrobactrum

Oligella

Onchocerca

Paecilomyces

Pantoea

Paracoccus

Paracoccidioides

Pasteurella

Pediococcus

Pedobacter

Penicillium

Phaeoannellomyces

Photobacterium

Plasmodium

Plesiomonas

Pneumocystis

Porphyromonas

Prevotella

Propionibacterium

Proteus

Providencia

Pseudomonas

Psychrobacter

Rahnella

Rhizobium

Rhizopus

Rhodococcus

Rhodotorula

Rickettsia

Roseomonas

Rothia

Salmonella

Sarcocystis

Scedosporium

Scopulariopsis

Schistosoma

Serratia

Shewanella

Shigella

Simonsiella

Sphingobacterium

Sphingomonas

Spirillum

Sporothrix

Staphylococcus

Streptococcus

Stenotrophomonas

Stomatococcus

Streptobacillus

Streptomyces

Strongyloides

Suttonella

Taenia

Tatumella

Tetragenococcus

Toxocara

Toxoplasma

Trabulsiella

Treponema

Trichoderma

Trichomonas

Trichophyton

Trichostrongylus

Trichuris

Tropheryma

Trypanosoma

Tsukamurella

Turicella

Ureaplasma

Vagococcus

Vibrio

Weeksella

Wuchereria

Yersinia

Yokenella

Zygomycete

Adenovirus

Alenquer virus

Alphavirus

Amapari virus

Andes virus

Arbovirus

Arenavirus

Astrovirus

Auravirus

Baculovirus

Banna virus

Banzi virus

Bayou virus

Bear Canyon virus

Bijou Bridge virus

BK virus

Black Creek Canal virus

Bloodland Lake virus

Bluetongue virus

Borna disease virus

Barmah Forest virus

Buffalopox virus

Bunyamwera virus

Bunyavirus

Bussuquara virus

Cabassou virus

Calabazo virus

Calchaqui virus

Calicivirus

Camberwell virus

Canarypox virus

Chagres virus

Chandipura virus

Changuinola virus

Chikungunya virus

Choriomeningitis virus

Colorado tick fever virus

Coltivirus

Convict Creek virus

Coronavirus

Cowpox virus

Coxsackievirus

Cupixi virus

Cytomegalovirus

Dekar bat virus

Dengue virus

Dobrava virus

Eastern equine encephalitis virus

Ebola virus

Echovirus

Ectromelia virus

Edge Hill virus

El Moro Canyon virus

Enterovirus

Epstein-Barr virus

Everglades virus

Eyach virus

Filovirus

Flavivirus

Flexal virus

Fort Morgan virus

Fowpox virus

GB virus

Grimsby virus

Guanarito virus

Hantaan virus

Hantavirus

Hendra virus

Hepatitis A virus

Hepatitis B virus

Hepatitis C virus

Hepatitis D virus

Hepatitis E virus

Hepatitis G virus

Herpes simplex virus

Herpesvirus

Highlands J virus

Human immunodeficiency virus

Human T-cell lymphotropic virus

Ilheus virus

Influenza virus

Ippy virus

Isfahan virus

Isla Vista virus

Jamestown Canyon virus

Japanese encephalitis virus

JC virus

Junin virus

Kadipiro virus

Kemerovo virus

Kokobera virus

Kunjin virus

Koutango virus

Kyasanur Forest disease virus

Kyzlagach virus

La Crosse virus

Lagos bat virus

Laguna Negra virus

Langat virus

Lassa virus

Latino virus

Lassavirus

Lebombo virus

Lipovnik virus

Leukemia virus

London virus

Lordsdale virus

Machupo virus

Marburg virus

Mayaro virus

Measles virus

MC virus

Mexico virus

Menangle virus

Mobala virus

Modoc virus

Mokola virus

Molluscum contagiosum virus

Monkeypox virus

Mucambo virus

Mumps virus

Murray Valley encephalitis virus

Nairovirus

Naples virus

Negishi virus

New York virus

Newcastle disease virus

Nipah virus

Norwalk virus

O'nyong-nyong virus

Oklahoma tick fever virus

Omsk hemorrhagic fever virus

Oliveros virus

Orbivirus

Orf virus

Orungovirus

Otofuke virus

Papillomavirus

Papova virus

Parainfluenza virus

Paramyxovirus

Paranavirus

Parvovirus

Phlebovirus

Pichinde virus

Piry virus

Pixuna virus

Plymouth virus

Poliovirus

Punta virus

Puumala virus

Rabies virus

Reovirus

Respiratory syncytial virus

Retrovirus

Rhabdovirus

Rhinovirus

Rift Valley fever virus

Rio Bravo virus

Rio Mamore virus

Rio Segundo virus

Rocio virus

Ross River virus

Rotavirus

Rubella virus

Sabia virus

Sandfly fever virus

Sapporo virus

Seadorna virus

Sealpox virus

Semliki Forest virus

Sendai virus

Seoul virus

Sepik virus

Sicilian virus

Simian virus

Sin Nombre virus

Sindbis virus

Six Gun City virus

Slamon River tick fever virus

Smallpox virus

Spondweni virus

Spumavirus

St. Louis encephalitis virus

Stockholm virus

Swinepox virus

Tacaribe virus

Tahyna virus

Tanapox virus

Thai virus

Tick-borne encephalitis virus

Tonate virus

Torovirus

Toscana virus

TT virus

Tula virus

Usutu virus

Vaccinia virus

Varicella-zoster virus

Variola virus

Venezuelan equine encephalitis virus

Wesselsbron virus

West Nile virus

Western equine encephalitis virus

Whataroa virus

Whitewater Arroyo virus

Yatapoxvirus

Yellow fever virus

Zika virus
