# Supplementary material for: A PubMed-Wide Associational Study of Infectious Diseases
Source: PLoS One. 2010 Mar 10;5(3):e9535. doi: 10.1371/journal.pone.0009535 (PMC2835740; doi:10.1371/journal.pone.0009535)
Supplement: Supplementary file 7 [file pone.0009535.s004.doc]

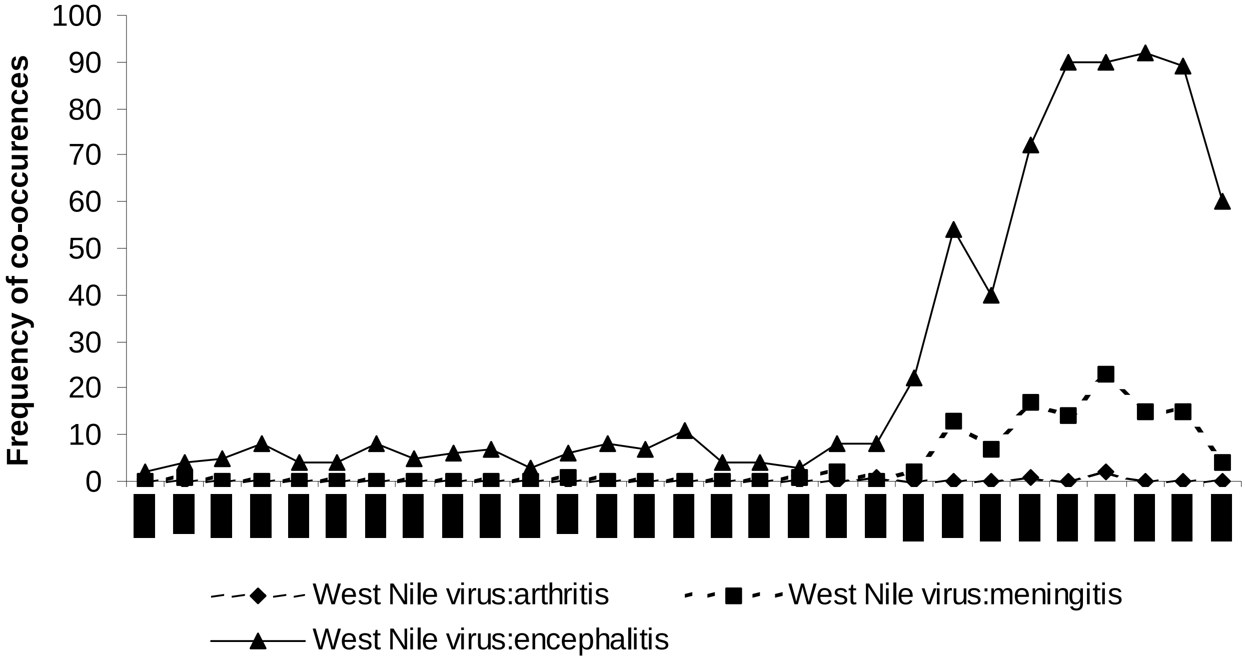
Frequency of co-occurences
100
90
80
70
60
50
40
30
20
10
0
West Nile virus:arthritis
West Nile virus:meningitis
West Nile virus:encephalitis
