# Supplementary material for: A PubMed-Wide Associational Study of Infectious Diseases
Source: PLoS One. 2010 Mar 10;5(3):e9535. doi: 10.1371/journal.pone.0009535 (PMC2835740; doi:10.1371/journal.pone.0009535)
Supplement: Supplementary file 8 [file pone.0009535.s006.doc]

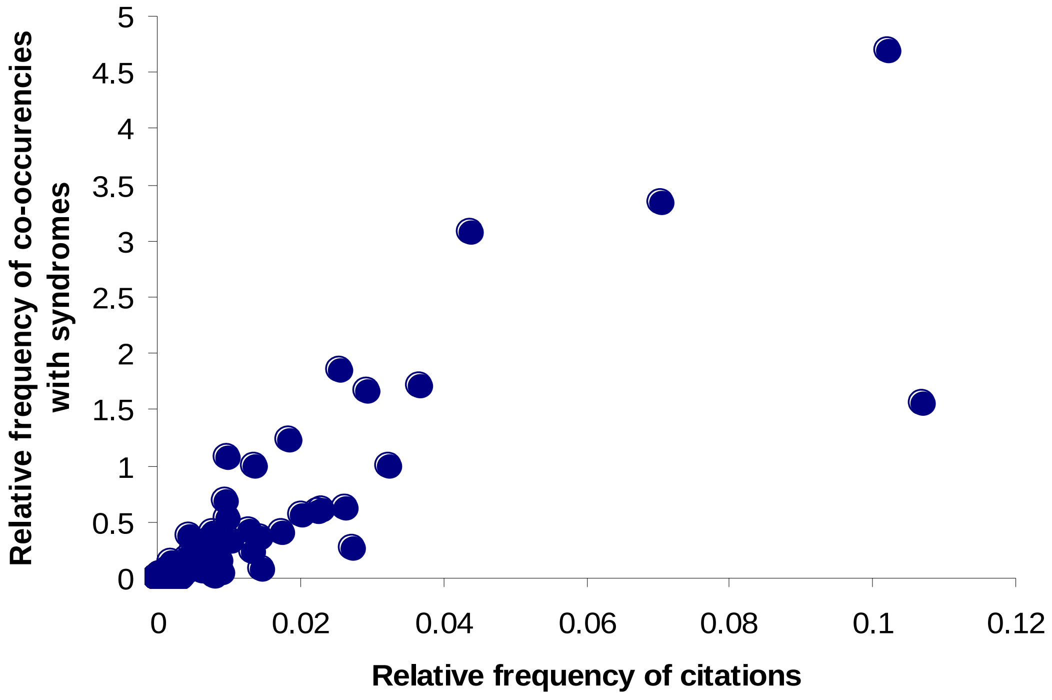
Relative frequency of co-occurencies with syndromes
5
4.5
4
3.5
3
2.5
2
1.5
1
0.5
0
0
0.02
0.04
0.06
0.08
0.1
0.12
Relative frequency of citations
